# Effect of exposed-to-air frequency of cryopreserved embryo on clinical outcomes of vitrified-warmed embryo transfer cycles: a retrospective analysis of 9,200 vitrified-warmed transfer cycles

**DOI:** 10.1186/s12884-023-05879-w

**Published:** 2023-08-17

**Authors:** Huan Zhang, Danna Ye, Yonggen Wu, Yan Li, Xuefeng Huang

**Affiliations:** Department of Reproductive Medical Center, The First Affiliated Hospital of Wenzhou Medical University, Fuxuexiang 96#, Wenzhou, 325000 Zhejiang China

**Keywords:** Embryo cryopreservation, EAF, Survival rate, Implantation, Live birth

## Abstract

**Background:**

Cryopreservation of embryos plays a major role in the in vitro fertilization (IVF)/intracytoplasmic sperm injection (ICSI) treatment. However, the storage condition of the cryopreserved embryo can change temporarily due to repeated retrieval of the embryo from the liquid nitrogen (LN_2_) tank during the practical application during cryopreservation. Whether the implantation potential of a cryopreserved embryo will be damaged when the cane containing it is temporarily exposed to air due to the transfer between the LN_2_ tank and LN_2_ container is yet to be elucidated. Also, whether the exposed-to-air frequency (EAF) of cryopreserved embryos influences the clinical outcomes is unclear.

**Objective:**

To investigate whether the EAF of cryopreserved embryo affects the clinical outcomes of vitrified-warmed embryo transfer.

**Methods:**

A total of 9200 vitrified-warmed embryo transfer cycles were included in this study. All cycles were divided into five groups according to different EAFs (2, 4, 6, 8, or ≥ 10). Post-warming survival rates and clinical outcomes, including implantation, clinical pregnancy and live birth rates were investigated. Kruskal–Wallis test and Pearson’s chi-squared tests were used to compare the patient characteristics and clinical outcomes among the five groups. Furthermore, multivariate logistic regression analyses were conducted to investigate the association between EAF and clinical outcomes.

**Results:**

No significant differences were observed in the positive HCG rate, implantation rate and live birth rate (*P* > 0.05) among five EAF groups with respect to D3 embryo, D5 blastocyst and D6 blastocyst. Post-warmed survival rate of D3 embryos (*P* = 0.015) differed significantly among the five EAF groups, but it was not EAF-dependent. Although clinical pregnancy was different among the five groups with respect to D5 blastocyst (*P* = 0.042), multivariate logistic regression analysis adjusted for confounding variables suggested that EAF did not adversely affect clinical pregnancy or live birth.

**Conclusion:**

These findings indicated that human vitrified embryos in the open system could be repeatedly retrieved from the LN_2_ tank without affecting the implantation potential of the embryo.

**Supplementary Information:**

The online version contains supplementary material available at 10.1186/s12884-023-05879-w.

## Background

The technique of embryo cryopreservation has been increasingly applied in clinical settings. It is beneficial for patients who produce large numbers of the embryo in a fresh cycle, reducing the risk of ovarian hyperstimulation syndrome and multiple pregnancy rates and increasing the cumulative pregnancy rate [1, 2]. Owing to the improvement in laboratory techniques, especially the emergence of vitrification during the last decade, vitrified-warmed embryo transfer (V-FET) has been widely used in several in vitro fertilization (IVF) centers. Unlike traditional slow freezing, vitrified-warming can decrease the ice crystal formation during vitrification or warming. Previous studies suggested that vitrification achieves a significantly higher embryo survival rate than slow freezing [3, 4]. Moreover, recent studies reported that the clinical outcomes of vitrified-warmed embryos transfer cycles were similar to or better than those of fresh embryos transfer cycles [3, 5, 6].

During the last decade, the number of vitrified embryos has been increasing gradually; however, some concerns were raised. Whether the viability of the embryo is stable during the long-term cryopreservation is uncertain. Previous studies yielded results on the effect of cryopreservation storage time on embryo survival and clinical outcomes. For traditional slow freezing, Tesart et al. demonstrated that embryo survival rate was decreased after several months of cryopreservation [7]. On the contrary, Riggs et al. revealed that the length of storage time has no influence on the clinical outcomes based on 11,768 cryopreserved human embryos [8]. For vitrification, some studies found storage time has no effect on the embryo survival rate and pregnancy outcomes when embryos were stored in LN_2_ for several years [9–13]. Conversely, some recent studies observed that storage time has a negative effect on pregnancy outcomes [14, 15]. These studies mainly focused on the effects of storage time on clinical outcomes. Nevertheless, a major issue about the storage condition of the embryo during cryopreservation has always been overlooked. During the practical process of cryopreservation, the canes loaded with Cryotop straws were repeatedly transferred between the LN_2_ container and canister of the LN_2_ tank to retrieve the cryotop straw for warming or storing it for cryopreservation; thus, the storage condition could be changed temporarily during the transfer of the canes. Therefore, embryos may experience transient warming or might experience a subtle injury during the transfer time from the LN_2_ container to the storage tank [4, 16, 17]. Whether this common but unnoticed practice of embryo transfer between containers with potential exposure, also defined as exposed-to-air frequency (EAF), could influence post-warmed survival rate and clinical outcomes are yet to be elucidated.

Furthermore, calculating the EAF using a laboratory paper record sheet is challenging and time-consuming. Fortunately, EAF can be recorded by a robust reproductive medicine management electronic system in our laboratory. This system could record every embryo straw positioned in a specifically appointed cane, canister and tank, the vitrifying and warming time, and the clinical information of subsequent V-FET. The data facilitated an easy and accurate calculation of the EAF of an embryo before warming for embryo transfer.

The present study aimed to investigate the effect of the EAF on the clinical outcomes of subsequent vitrified-warmed embryo transfer and whether limits should be set for the number of straws in a cane and the number of canes in a canister.

## Materials and methods

### Study design

This retrospective study used extracted data from our reproductive medicine management electronic system, including 9,200 vitrified-warmed transfer cycles (3,313 D3 V-FET cycles, 4,530 D5 V-FET cycles and 1,357 D6 V-FET cycles) between January 2011 and December 2016. The V-FET cycles with thin endometrial thickness (˂ 7 mm) on the embryo transfer day were excluded. In the present study, five EAF groups were defined: group 1, EAF is 2; group 2, EAF is 4; group 3, EAF is 6; group 4, EAF is 8; group 5, EAF is ≥ 10.

### Ovarian stimulation and vitrified embryo quality

Different controlled ovarian stimulation protocols were selected for patients in fresh cycles due to the diversity of patient characteristics. Ultrasonography-guided oocyte retrieval was performed 36 h after human chorionic gonadotropin (HCG, Livzon, China) injection. After 3–4 h, oocyte insemination was achieved by intracytoplasmic sperm injection or conventional in vitro fertilization according to clinical indications. About 16–18 h post-insemination or injection, fertilization was confirmed based on the presence of two pronuclei and two polar bodies. In the morning of day 3, the morphology of the embryos was graded according to the Istanbul consensus [18]. The two best embryos were considered for transfer or cryopreservation according to the clinical indications: 1) if the patient has the risk of ovarian hyperstimulation syndrome, endometrial thickness ˂ 7 mm, hydrosalpinx, or other conditions that the clinician considered not suitable for transfer, the best two embryos were vitrified; 2) Otherwise, the best two embryos were transferred. The surplus embryos were commonly transferred from cleavage culture medium to blastocyst culture medium for further culture. The blastocysts were evaluated on days 5 and 6 according to Gardner scoring [18]. Blastocyst graded ≥ 3 BC was frozen by vitrification.

### Vitrification, warming and cryopreservation protocols

Vitrification and warming were carried out according to the method described by Kuwayama [19] using Vitrification and Warming kits (Kitazato Biopharma, Shizuoka, Japan). The vitrified embryos were loaded into an open system. Briefly, for vitrification, embryos were first incubated into an equilibration buffer at room temperature for 10 min and then transferred into a vitrification solution for 30–60 s. Then, embryos were loaded on the Cryotop straw and plunged into fresh LN_2_ in an open container. Up to 9 cryotop straws were placed in one cane, up to 16 canes were put in one canister, and about 6–10 canisters were put in one tank. For warming, the cryotop straw was taken out of LN_2_ and quickly submerged into the warming solution at 37 ℃. After 1 min, the embryos were transferred into dilution solutions at room temperature for 3 min. Then, the embryos were placed in wash solution 1 at room temperature for 5 min. Last, the embryos were washed with wash solution 2 at 37 ℃ for 5 min. The warmed embryos were evaluated again before transfer. In early cleavage embryo, survival indicated embryo with ≥ 50% intact blastomeres [10]. The blastocysts were evaluated as described by Allen et al. [20], and survival means blastocyst with > 50% cell survival. The embryo (blastocyst) survival rate was defined as the number of survived embryos (blastocysts) / the number of thawed embryos (blastocysts) × 100% [21].

### Embryo transfer and outcomes

Hormone replacement or nature cycle was conducted for endometrial preparation. On day 3 or 5, embryo transfer was conducted under ultrasound guidance. Serum HCG was measured 14 days after embryo transfer.

Clinical pregnancy was defined as the presence of at least one gestational sac with fetal heartbeat on ultrasound scan 4–5 weeks after embryo transfer. The implantation rate was calculated as follows: number of gestational sacs / the number of transferred embryos. Live birth was defined as the delivery of any neonate who was ≥ 28 weeks of gestation after embryo transfer [22].

### Calculation of EAF

The consumables and equipment for cryopreservation included Cryotop straw loaded with the embryo(s) (Kitazato Biopharma), cane (loaded with straws, Minitube, 16981/01361, Slovakia), LN_2_ container (a Styrofoam box) for vitrification or warming, canister for storage of canes, and LN_2_ tank (MVE, XC 47/11–10, USA). The EAF of an embryo was equal to that of the Cryotop straw loading with this embryo. Overall, the EAF of a straw was decided by the frequency of the cane contained with the straw in and out of the LN_2_ tank. The EAF was calculated based on the following criteria: [1] After vitrification, the straw containing vitrified embryo(s) was inserted into a cane in a LN_2_ container, then the cane was taken out of the LN_2_ container and plunged into a canister in a LN_2_ tank, this was considered one time to EAF of this straw; if the cane was loaded with another straw before, the other straws in this cane were marked as two times to their EAF (one time for taken out of the tank and one time for placing it back into the tank); [2] Before warming, the cane containing a straw was taken out from a canister in the LN_2_ tank into a LN_2_ container, one time is added to the EAF of this straw; moreover, other straws in this cane were deemed as two times added to their EAF (one time for taking out of tank and one time for putting back into the tank); [3] If more than one straw was taken out or put into one cane simultaneously, EAF was added as the rule of one straw. The duration of every cane transferred between LN_2_ container and tank was supposed to be similar, as experienced senior embryologists performed all the operations (detailed description about the calculation of EAF can refer to the Supplementary file named “How to calculate the EAF”). The EAF of a straw could be counted because the vitrifying and warming times and the position in the specially appointed cane, canister and tank of every straw were recorded in real-time by the reproductive medicine electronic system, which was developed by information department of our hospital. The operation of reproductive medicine electronic system software was relied on computer server of our hospital.

### Statistical analysis

Continuous variables were described as mean with standard deviation (x ± SD) and analyzed by Kruskal-Wallis test. Pair-wise comparisons were performed by Bonferroni method. Categorical variables were compared using Pearson’s chi-squared test. Multivariate logistic regression analyses were used to evaluate the association between clinical outcomes and EAF. *P* ˂ 0.05 was considered statistically significant. All statistical analyses were performed using SPSS version 20.0 (SPSS Inc, USA).

## Results

Herein, 9200 V-FET cycles were evaluated, including 3,313 D3 V-FET cycles (7054 D3 embryos), 4,530 D5 V-FET cycles (7761 D5 blastocysts) and 1,357 D6 V-FET cycles (2129 D6 blastocysts). D3, D5 and D6 V-FET cycles were analyzed separately due to the varied features in the embryo development stage. Patient characteristics and clinical outcomes of different EAF groups for D3, D5 and D6 V-FET cycles are listed in Tables 1, 2 and 3, respectively.

**Table 1:** Patient characteristics and clinical outcomes of EAF in vitrified-warmed day 3 embryo transfer cycles

Characteristics	Group 1	Group 2	Group 3	Group 4	Group 5	*P*
EAF	2	4	6	8	≥ 10	
No. of cycles	974	894	611	350	484	
**Storage time (days)**	83.10 ± 109.93^a,b,c,d^	103.58 ± 80.35^a,e,f,g,^	153.96 ± 156.64^b,e,h,i^	196.31 ± 204.71^c,f,h,j^	300.63 ± 306.47^d,g,i,j^	** < 0.001**
Age of female patients at ET (years)	32.42 ± 5.44	32.60 ± 5.29	32.53 ± 5.31	32.45 ± 4.76	33.00 ± 5.46	0.324
Age of female patients at VIT (years)	32.19 ± 5.45	32.31 ± 5.29	32.11 ± 5.28	31.91 ± 4.80	32.17 ± 5.44	0.914
BMI (kg m^2^)	21.81 ± 3.33	21.63 ± 2.90	21.75 ± 3.12	21.59 ± 2.84	21.68 ± 2.95	0.977
Duration of infertility (years)	3.89 ± 2.84	3.87 ± 3.06	4.05 ± 3.06	3.61 ± 2.64	3.77 ± 2.99	0.185
Endometrial thickness (mm)	9.29 ± 1.48	9.16 ± 1.46	9.18 ± 1.48	9.12 ± 1.52	9.12 ± 1.51	0.070
No. of embryos warmed	2.13 ± 0.52	2.14 ± 0.49	2.11 ± 0.46	2.12 ± 0.44	2.15 ± 0.47	0.477
No. of transferred embryos	2.05 ± 0.47	2.06 ± 0.44	2.06 ± 0.42	2.09 ± 0.45	2.09 ± 0.47	0.391
**ET with high quality embryos rate, % (n)**	65.4 (1308)^a^	63.4 (1169)	64.0 (805)	63.2 (463)	59.3 (601)^a^	**0.028**
**Survival rate, % (n)**	96.6 (2000)^a^	96.5 (1844)^b^	97.7 (1258)	98.7 (733)^a,b^	97.1 (1013)	**0.015**
Implantation rate, % (n)	27.5 (549)	25.9 (477)	27.7 (349)	24.8 (182)	23.8 (241)	0.136
Positive HCG rate, % (n)	50.3 (490)	49.3 (441)	52.9 (323)	47.7 (167)	48.6 (235)	0.503
Clinical pregnancy rate, % (n)	42.6 (415)	41.3 (369)	44.8 (274)	39.1 (137)	38.4 (186)	0.203
Live birth rate, % (n)	34.4 (335)	34.5 (308)	37.6 (230)	31.1 (109)	31.4 (152)	0.148

Values with the same superscript letter did differ significantly in pare-wise comparisons

*VIT* Vitrification

**Table 2:** Patient characteristics and clinical outcomes of different EAFs in vitrified-warmed day 5 blastocyst transfer cycles

Characteristics	Group 1	Group 2	Group 3	Group 4	Group 5	*P*
EAF	2	4	6	8	≥ 10	
No. of cycles	1131	1134	811	523	931	
**Storage time (days)**	83.42 ± 123.29^a,b,c,d^	121.91 ± 174.16^a,e,f,g^	168.58 ± 207.67^b,e,h,i^	240.11 ± 307.52^c,f,h,j^	433.92 ± 434.32^d,g,i,j^	** < 0.001**
**Age of female patients at ET (years)**	30.68 ± 4.05^a^	30.73 ± 4.02^b^	30.80 ± 4.16^c^	31.38 ± 4.33	31.58 ± 3.91^a,b,c^	** < 0.001**
Age of female patients at VIT (years)	30.45 ± 4.04	30.39 ± 3.98	30.34 ± 4.12	30.72 ± 4.23	30.39 ± 3.91	0.716
BMI (kg m^2^)	21.31 ± 2.81	21.45 ± 3.24	21.19 ± 2.99	21.55 ± 3.23	21.31 ± 2.80	0.454
Duration of infertility (years)	4.03 ± 2.94	3.81 ± 2.77	3.70 ± 2.67	4.10 ± 3.10	4.02 ± 2.90	0.060
Endometrial thickness (mm)	9.06 ± 1.40	9.09 ± 1.43	9.08 ± 2.23	9.15 ± 1.54	9.01 ± 1.46	0.357
**No. of embryos warmed**	1.77 ± 0.45^a,b,c,d^	1.71 ± 0.46^a^	1.70 ± 0.47^b^	1.68 ± 0.47^c^	1.67 ± 0.49^d^	** < 0.001**
**No. of embryos transferred**	1.75 ± 0.43^a,b,c,d^	1.70 ± 0.46^a^	1.69 ± 0.46^b^	1.67 ± 0.47^c^	1.66 ± 0.48^d^	** < 0.001**
**ET with high quality embryos, % (n)**	67.6 (1339)^a^	67.6 (1300)^b^	65.8 (901)	64.9 (567)	62.7 (967)^a,b^	**0.015**
Survival rate, % (n)	98.7 (1981)	99.4 (1924)	99.1 (1369)	99.3 (873)	99.0 (1543)	0.162
Implantation rate, % (n)	43.2 (855)	39.7 (763)	40.5 (554)	40.3 (352)	42.6 (658)	0.145
Positive HCG rate, % (n)	64.6 (731)	61.5 (697)	63.1 (512)	60.6 (317)	64.6 (601)	0.322
**Clinical pregnancy rate, % (n)**	57.4 (649)	52.7 (598)	53.0 (430)	50.7 (265)	56.1 (522)	**0.042**
Live birth rate, % (n)	48.7 (551)	44.7 (507)	43.5 (353)	44.4 (232)	46.3 (431)	0.173

Values with the same superscript letter did differ significantly in pare-wise comparisons

*VIT* Vitrification

**Table 3:** Patient characteristics and clinical outcomes of different EAFs in vitrified-warmed day 6 blastocyst transfer cycles

Characteristics	Group 1	Group 2	Group 3	Group 4	Group 5	*P*
EAF	2	4	6	8	≥ 10	
No. of cycles	329	330	248	172	278	
**Storage time (days)**	99.86 ± 143.61^a,b,c,d^	158.71 ± 228.96^a,e,f,g^	184.33 ± 201.48^b,e,h,i^	272.79 ± 299.21^c,f,h,j^	491.22 ± 487.26^d,g,i,j^	** < 0.001**
Age of female patients at ET (years)	31.55 ± 4.14	31.58 ± 4.23	31.24 ± 4.01	31.51 ± 4.04	32.24 ± 4.13	0.141
Age of female patients at VIT (years)	31.27 ± 4.12	31.14 ± 4.24	30.74 ± 3.99	30.77 ± 4.12	30.90 ± 4.05	0.566
BMI (kg/m^2^)	21.34 ± 3.05	21.30 ± 2.74	21.27 ± 3.34	21.14 ± 2.91	21.64 ± 3.46	0.379
Duration of infertility (years)	4.09 ± 2.90	4.28 ± 3.05	3.82 ± 2.89	3.82 ± 2.80	4.18 ± 3.14	0.268
Endometrial thickness (mm)	9.22 ± 1.48	8.93 ± 1.35	8.87 ± 1.40	9.06 ± 1.49	8.98 ± 1.49	0.090
No. of embryos warmed	1.59 ± 0.52	1.62 ± 0.55	1.54 ± 0.50	1.56 ± 0.53	1.51 ± 0.56	0.114
No. of embryos transferred	1.57 ± 0.50	1.58 ± 0.50	1.51 ± 0.50	1.54 ± 0.51	1.49 ± 0.50	0.150
ET with high quality embryos % (n)	58.1 (299)	57.2 (298)	56.0 (210)	53.6 (142)	55.9 (231)	0.805
Survival rate, % (n)	98.7 (515)	97.4 (521)	98.2 (375)	98.5 (265)	98.1 (413)	0.622
Implantation rate, % (n)	29.7 (153)	30.3 (158)	34.7 (130)	28.7 (76)	29.1 (120)	0.399
Positive HCG rate, % (n)	49.2 (162)	51.5 (170)	53.6 (133)	51.7 (89)	51.4 (143)	0.892
Clinical pregnancy rate, % (n)	39.5 (130)	39.7 (131)	44.8 (111)	40.1 (69)	37.4 (104)	0.534
Live birth rate, % (n)	33.7 (111)	30.9 (102)	33.9 (84)	30.8 (53)	28.4 (79)	0.643

Values with the same superscript letter did differ significantly in pare-wise comparisons

*VIT* Vitrification

The storage time was increased from group 1 to group 5 (Tables 1, 2, and 3). Table 1 shows that ET with high-quality embryos and survival rate differed among the five groups, while the other variables, including implantation rate, positive HCG rate, clinical pregnancy rate and the live birth rate, did not differ significantly among the five groups.

Table 2 shows that the age of female patients at ET, ET with high-quality embryos rate, number of embryos warmed and number of transferred embryos differed among the five groups. Surprisingly, the clinical pregnancy rate was different among the five groups. In addition, other variables, including the embryo survival rate, positive HCG rate, implantation rate and live birth rate, did not differ significantly among the five groups.

In Table 3, no difference was observed in patient characteristics and clinical outcomes except storage time among the five groups.

In Table 4, multivariate logistic regression analysis for clinical pregnancy and live birth was conducted, including the following variables: EAF, storage time, age of female patients at ET, age of male patients at vitrification (VIT), number of ET attempts, number of embryos transferred, number of high-quality embryos transferred, type of infertility, number of oocytes retrieved, number of high-quality embryos on day3 and controlled ovarian hyperstimulation (COH) protocol in fresh cycles. The results demonstrated that EAF did not adversely affect clinical pregnancy or live birth with respect to D3 embryo and D5 and D6 blastocyst.

**Table 4:** Multivariate logistic regression analysis of EAF for clinical pregnancy and live birth, adjusting for potential confounders

Embryo frozen day	Variables	Clinical pregnancy		Live birth	
		OR (95% CI)	*P*	OR (95% CI)	*P*
D3	EAF		0.232		0.124
	2	reference		reference	
	4	0.962 (0.793–1.167)	0.692	1.025 (0.838–1.254)	0.808
	6	1.121 (0.902–1.393)	0.303	1.194 (0.953–1.496)	0.123
	8	0.844 (0.647–1.102)	0.214	0.835 (0.631–1.104)	0.206
	≥ 10	0.867 (0.672–1.119)	0.274	0.898 (0.688–1.173)	0.431
D5	EAF		0.128		0.392
	2	reference		reference	
	4	0.867 (0.730–1.030)	0.103	0.897 (0.755–1.065)	0.213
	6	0.888 (0.735–1.073)	0.218	0.865 (0.716–1.045)	0.134
	8	0.838 (0.673–1.045)	0.116	0.935 (0.750–1.166)	0.551
	≥ 10	1.048 (0.856–1.282)	0.652	1.018 (0.833–1.245)	0.859
D6	EAF		0.382		0.811
	2	reference		reference	
	4	1.010 (0.730–1.397)	0.954	0.888 (0.632–1.248)	0.494
	6	1.348 (0.950–1.913)	0.094	1.067 (0.741–1.536)	0.727
	8	1.085 (0.727–1.620)	0.690	0.928 (0.609–1.413)	0.727
	≥ 10	0.967 (0.661–1.414)	0.862	0.859 (0.574–1.287)	0.462

*CI* Confidence interval, *OR* Odds ratio

Adjusted for confounders (storage time, age of female patients at ET, age of male patients at VIT, No. of ET attempts, No. of embryos transferred, No. of high-quality embryo transferred, No. of oocytes retrieved, No. of high-quality embryo on day 3, COH protocol, type of infertility)

## Discussion

In the present study, no significant differences were observed in positive HCG rate, clinical pregnancy rate and live birth rate among the five EAF groups with respect to D3 embryo and D6 blastocyst. Although univariate analysis found that the clinical pregnancy was different among the five groups regarding D5 blastocyst, multivariate logistic regression analysis adjusted for confounding variables suggested that EAF did not adversely affect clinical pregnancy or live birth. These results indicated that vitrified human embryos in an open system could be repeatedly retrieved from the liquid nitrogen tank without affecting the implantation potential of the embryo.

Furthermore, we observed that the storage time increased from group 1 to group 5, suggesting a positive correlation between EAF and storage time. Commonly, the longer the embryos were stored in the LN_2_ tank, the higher the probability that other cryotop straws in the same cane would be taken out for warming or put in for cryopreservation. Hitherto, whether storage time influences the clinical outcomes remains controversial. Typically, the embryos receive 0.1 radiation dose/year, which is accumulated over time during cryopreservation. Animal models had demonstrated that the mouse embryo survival rate was not decreased even when the radiation dose equivalent to 2,000 years of normal background radiation levels was administered [23]. However, the model has limited application values because of the significant differences between human and animal physiology. In human studies, the effect of storage time yielded contradictory results. For slow freezing, Testart et al. reported that the storage time has a negative effect on the embryo survival rate, but the limitation of the study was that it did not control for pre-freeze embryo morphology [7]. Riggs et al. presented a larger analysis and found that the storage time did not affect the clinical outcomes [8]. Nonetheless, the freezing method of these two differed from that in the present study; the vitrified embryo would interact with the reactive oxygen or nitrogen compounds in the liquid nitrogen when using an open system. For vitrification, Li et al. found that the embryo survival rate, pregnancy and obstetric outcomes were not related to storage time [10]. Wirleitner et al. demonstrated that the cryopreservation storage time has no effect on blastocyst survival and pregnancy outcomes when these blastocysts are stored in the LN_2_ tank for up to 6 years [11]. In contrast, Cui et al. showed that the live birth and clinical pregnancy rates decreased significantly when the storage time exceeded 5 years [24]; however, the sample size of these three studies was small. Recently, two large retrospective analyses showed that the prolonged storage time was negatively associated with pregnancy and live birth [14, 15]. Nevertheless, there were some limitations in their study. There was a weakness of the study by Zhang et al. [14] which did not analyze D5 blastocyst or D6 blastocyst separately, because transfers of D5 blastocyst present much higher pregnancy and live birth rate than the transfers of D6 blastocyst [25]. In the study by Li et al. [15], the average age of the patients was increased in a prolonged storage time group.

During cryopreservation, the embryos were stored in liquid nitrogen at −196 °C; this temperature is below the glass transition temperature (Tg), ranging from −126 to −121°C [17]. The macromolecular diffusion was inhibited, and thermo-mechanical stresses were reduced under Tg [26], limiting the chemical changes in the vitrified embryo. In our laboratory, we monitor the LN_2_ level of the LN_2_ tank twice a week and keep the tank full with LN_2_, avoiding temperature change that might be caused by LN_2_ evaporation [27, 28]. However, repeated retrieval of the embryo from the LN_2_ tank in the process of practical application during cryopreservation may alter the temperature. Sansinena et al. reported that the process of devitrification which could cause cell damage was initiated when the temperature reaches −109 °C [17]. Presently, little is known about whether EAF affects the environment around the embryo and thus affects the implantation potential of the embryo. In the present study, we demonstrated that EAF has no influence on clinical outcomes. To the best of our knowledge, this is the first study to investigate the correlation between EAF and clinical outcomes.

Typically, the clinical outcomes of V-FET cycles vary when the embryos at different stages are transferred [29]. Therefore, D3 embryos, D5 blastocysts and D6 blastocysts should be analyzed respectively. In the present study, we observed that the high-quality rate of transferred embryos was decreased in D3 and D5 V-FET cycles. Although no significant difference was detected in D6 V-FET cycles, the high-quality rate of transferred embryos was high in the lower EAF groups, which might be ascribed to our embryo transfer policy. In our center, we choose the cryotop straws with better quality embryos for warming when one patient had more than two cryotop straws with vitrified embryos; if implantation failure occurred, a worse one would be warmed, resulting in the cryotop straws with better quality embryos experiencing less exposure.

The survival rate of the D3 embryo varied among groups (*P *= 0.015), and it was difficult to conclude that EAF undermined the post-thaw survival of the embryo as the survival rate was not EAF-dependent, which might be attributed to random factors. The number of embryos warmed and the number of embryos transferred in D5 V-FET cycles showed a declining trend from group 1 to 5, which was not observed in D3 and D6 V-FET cycles, because one or two D5 blastocysts were placed on each cryotop straw, while two D3 embryos or two D6 blastocysts were placed on each cryotop conventionally in our center. In the clinical process, the patient preferred two blastocysts for transplantation, leading the cryotop straws with two D5 blastocysts to be warmed first, such that the embryos warmed and transferred per cycle decreased from group 1 to group 5. Although we observed different clinical pregnancy rates in D5 blastocysts among the five groups, multivariate logistic regression analysis adjusted for confounding variables did not exhibit any correlation between the clinical pregnancy rate and EAF.

Nevertheless, the present study has some major limitations. First, the present study is the lack of data on obstetric and neonatal outcomes, which should be collected in future studies. Second, the present study is a retrospective design. In order to reduce this defect, we analyzed the data, including 9200 V-FET cycles. The third limitation is that EAF is calculated by the number of exposed-to-air during the procedure rather than duration time of exposed-to-air. Because there is person (embryologist) variation in the time taken for every procedure.

## Conclusions

When the open system is used, vitrified embryos stored in the LN_2_ tank can be briefly taken out of the LN_2_ tank without affecting the clinical pregnancy and live birth. However, the long-term follow-up of the children born is required in future studies.

### Supplementary Information

**Additional file 1.**

## Data Availability

The datasets used and analyzed during the current study available from the corresponding author on reasonable request.

## Abbreviations

IVF: In vitro fertilization
ICSI: Intracytoplasmic sperm injection.
LN_2_: Liquid nitrogen
EAF: Exposed-to-air frequency
HCG: Human chorionic gonadotropin
V- FET: Vitrified-warmed embryo transfer
