# Supplementary material for: Effect of exposed-to-air frequency of cryopreserved embryo on clinical outcomes of vitrified-warmed embryo transfer cycles: a retrospective analysis of 9,200 vitrified-warmed transfer cycles
Source: BMC Pregnancy Childbirth. 2023 Aug 17;23:590. doi: 10.1186/s12884-023-05879-w (PMC10433674; doi:10.1186/s12884-023-05879-w)
Supplement: Supplementary file 1 — Additional file 1. [file 12884_2023_5879_MOESM1_ESM.docx]

Pictures show how to calculate the EAF


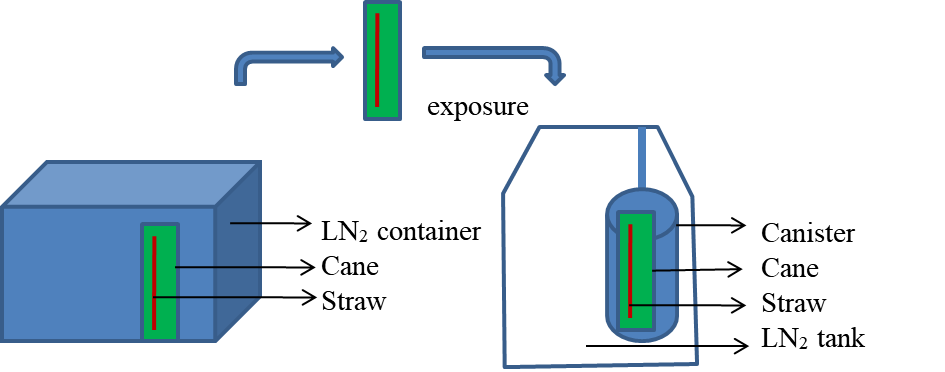


Fig 1 After vitrification, the straw containing vitrified embryo(s) was inserted into a cane in a LN_2_ container, then the cane was taken out of the LN_2_ container and plunged into a canister in a LN_2_ tank, this was considered one time to EAF of this straw.


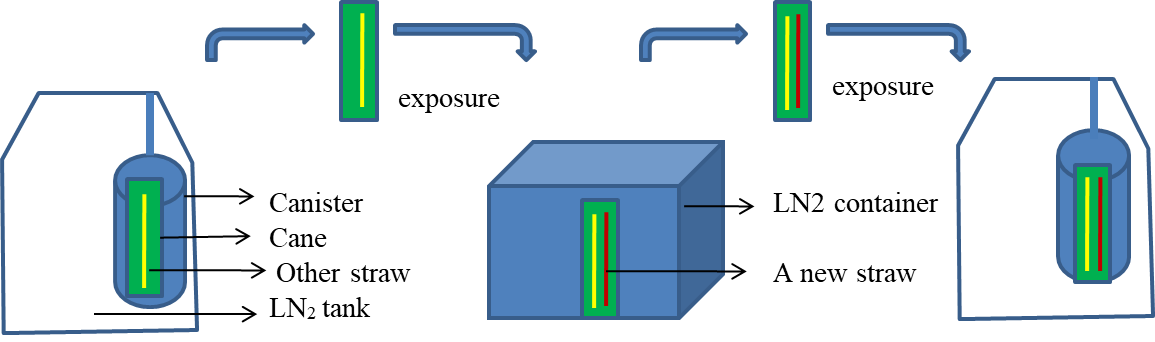


Fig 2 During the cryopreservation, once a new cryotop straw needs to be loaded into a cane filled with other straws already, the cane would be transferred out of canister into a LN_2_ container, after putting the new cryotop straw into the cane, the cane would be moved back into the LN_2_ tank, other straws in this cane were marked as two times to their EAF (one time for taken out of the tank and one time for placing it back into the tank).


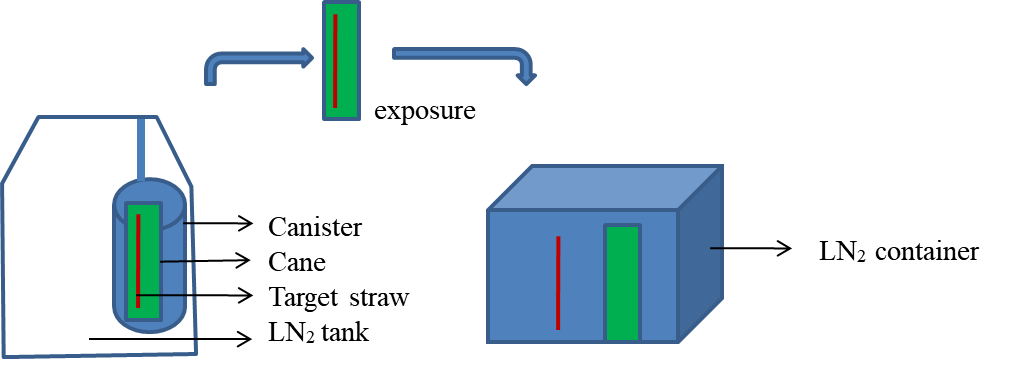


Fig 3 Before warming, the cane containing target straw for warming was transferred from canister of LN_2_ tank to an container filled with LN_2_, one time is added to the EAF of this target straw.


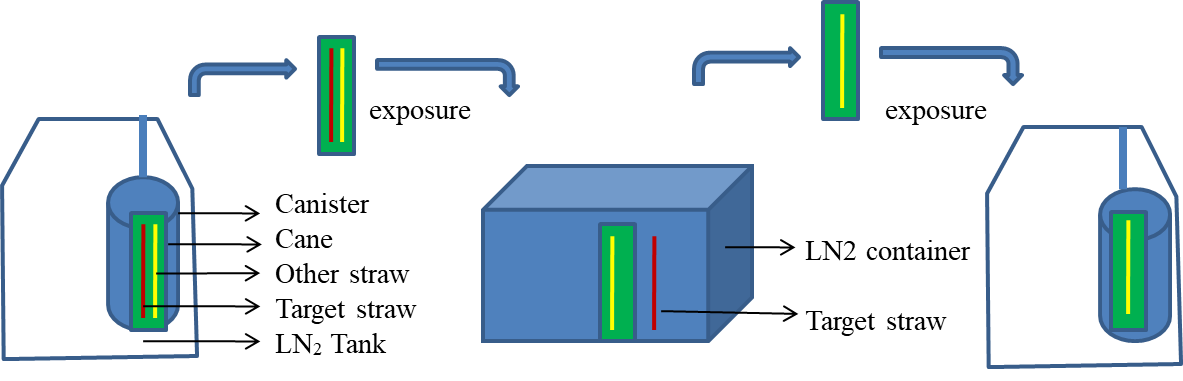


Fig 4 Once a target cryotop straw needs to be warmed, other straws stored in the same cane would be transferred from canister to a LN_2_ container together, and after putting the target cryotop straw into LN_2_ container, the cane would be moved back into the LN_2_ tank. Other straws in this cane were deemed as two times added to their EAF (one time for taking out of tank and one time for putting back into the tank)
